# Clinical outcomes of definitive whole pelvic radiotherapy for clinical lymph node metastatic prostate cancer

**DOI:** 10.1002/cam4.2985

**Published:** 2020-08-04

**Authors:** Keisuke Tsuchida, Koji Inaba, Tairo Kashihara, Naoya Murakami, Kae Okuma, Kana Takahashi, Hiroshi Igaki, Yuko Nakayama, Aiko Maejima, Yasuo Shinoda, Yoshiyuki Matsui, Motokiyo Komiyama, Hiroyuki Fujimoto, Yoshinori Ito, Minako Sumi, Takashi Nakano, Jun Itami

**Affiliations:** ^1^ Department of Radiation Oncology National Cancer Center Hospital Tokyo Japan; ^2^ Department of Radiation Oncology Gunma University Graduate School of Medicine Maebashi Japan; ^3^ Department of Urology National Cancer Center Hospital Tokyo Japan; ^4^ Department of Radiation Oncology Showa University School of Medicine Tokyo Japan; ^5^ Radiation Oncology Department Cancer Institute Hospital Japanese Foundation for Cancer Research Tokyo Japan

**Keywords:** dose escalation, IMRT, lymph node metastases, prostate cancer, radiotherapy

## Abstract

**Background:**

In this study, we aim to present the clinical outcomes of radiotherapy (RT) in clinical pelvic lymph node‐positive prostate cancer (cN1) patients. We also analyze the prognostic factors with focus on RT dose escalation to metastatic lymph nodes (LN).

**Methods:**

We retrospectively analyzed the data from cN1 patients who were treated with definitive RT and androgen deprivation therapy (ADT) between June 2004 and February 2016. All patients received localized irradiation to the prostate region and whole pelvis irradiation. Some patients received intensity‐modulated radiation therapy with RT dose escalation to metastatic LN. Univariate analyses using log‐rank test were performed to find prognostic factors between patient subgroups.

**Results:**

Fifty‐one consecutive patients were identified. The median follow‐up period for all patients was 88 (range 20‐157) months. Primary Gleason pattern and LN RT dose were statistically significant prognostic factors for relapse‐free survival (RFS) and distant metastasis‐free survival (DMFS). Especially, RT dose escalation (60 Gy or more) to metastatic LN significantly improved RFS and DMFS compared with standard dose RT (4‐year RFS 90.6% vs 82.1%, 7‐year RFS 90.6% vs 58.0%, *P* = .015; 4‐year DMFS 90.6% vs 82.1%, 7‐year DMFS 90.6% vs 62.8%, *P* = .023). The following factors were all statistically significant for biochemical relapse‐free survival (BRFS): T stage, LN RT dose, local RT dose, and ADT duration period. Any significantly different toxicity was not seen for each LN or local RT dose except for the incident rate of grade 2 or more acute urinary retention, which was significantly higher in the higher LN RT dose (60 Gy or more) group by the Chi‐square test.

**Conclusions:**

RT dose escalation to metastatic LN in cN1 patients improves BRFS, RFS, and DMFS at 4 and 7 years, without increasing severe adverse events.

## Graphical abstract

In this retrospective study, we aim to present the clinical outcomes of radiotherapy (RT) in clinical pelvic lymph node‐positive prostate cancer (cN1) patients. We also analyze the prognostic factors with focus on RT dose escalation to metastatic lymph node (LN). RT dose escalation to metastatic LN in cN1 patients improves relapse‐free survival (RFS) and distant metastasis‐free survival (DMFS) at 4 and 7 years, without increasing severe adverse events.

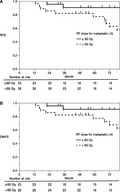

**Abbreviations**
ADT: androgen deprivation therapy
BRFS: biochemical relapse‐free survival
CSS: cause‐specific survival
CT: computed tomography
DMFS: distant metastasis‐free survival
GP: Gleason pattern
GS: Gleason score
IMRT: intensity‐modulated radiotherapy
LN: lymph node
MRI: magnetic resonance imaging
NCCN: National Comprehensive Cancer Network
OS: overall survival
PSA: prostate‐specific antigen
RFS: relapse‐free survival
RP: radical prostatectomy
RT: radiotherapy

## 1 INTRODUCTION

The benefits of adding radiotherapy (RT) to clinical pelvic lymph node (LN)‐positive prostate cancer (cN1) has been confirmed. Some clinicians consider pelvic LN metastases to be a marker of systemic disease, whereas others consider it to be regional and an indication for definitive local treatment. Therefore, the National Comprehensive Cancer Network (NCCN) guidelines include treatment options of both palliation with androgen deprivation therapy (ADT) ± abiraterone and prednisone, and definitive intense therapy with RT and ADT.^1^

Recently, some retrospective and database studies have demonstrated the benefits of adding definitive local treatment including radical prostatectomy (RP) and/or RT to ADT for cN1 patients.^2^, ^3^, ^4^, ^5^, ^6^, ^7^, ^8^, ^9^ However, the appropriate radiation field and its dose have not been well determined. Some studies include pelvic nodal area^4^, ^7^, ^9^ and others include only the prostate and seminal vesicles.^2^, ^3^

In our institution, we have performed RT to the whole pelvic nodal area, prostate, and seminal vesicles for cN1 patients. From 2008, we have been using intensity‐modulated RT (IMRT) to enable brief dose escalation for positive pelvic nodes of cN1 patients. In the present study, we present our clinical experience and outcomes of RT in cN1 patients and analyze the prognostic factors, especially dose escalation to positive pelvic nodes.

## 2 MATERIALS AND METHODS

### 2.1 Patients

Data from cN1 patients who had undergone definitive RT and ADT between June 2004 and February 2016 were analyzed retrospectively. The clinical stage was determined comprehensively using digital rectal examination, magnetic resonance imaging (MRI), computed tomography (CT), transrectal ultrasound, and bone scintigraphy. Patients with enlarged lymph nodes (LN) of the true pelvis on CT or MRI were diagnosed as cN1. Patients with distant metastasis including nonregional LN metastasis were excluded.

### 2.2 Radiotherapy

External beam RT was performed by 3D conformal radiotherapy (3DCRT) or IMRT using linear accelerator (Clinac iX or TrueBeam, Varian Medical Systems). Since 2008, IMRT for cN1 has been applied in our institution. Image‐guided radiotherapy was performed before each irradiation. The methods used for 3DCRT and IMRT are described in detail previously.^10^, ^11^

All patients received whole pelvis irradiation of 40‐46 Gy followed by or before localized irradiation of 72 Gy or 78 Gy to the prostate region in a daily fractional dose of 2 Gy. After the initiation of applying IMRT, some patients received IMRT with RT dose escalation to metastatic LN.

In whole pelvic irradiation, the clinical target volume of the whole pelvis (CTV‐WP) included the whole prostate and seminal vesicles, and the pelvic LN regions—namely the bilateral common iliac, presacral, internal iliac, obturator, and external iliac. The planning target volume of the whole pelvis was defined as CTV‐WP with a 4‐ to 10‐mm margin. In localized irradiation, the prostate and the bilateral seminal vesicles were defined as CTV‐local, with inclusion of the extraprostatic tumor extension, if present. PTV‐local in 3DCRT was defined as CTV‐local plus 6‐ to 10‐mm margins and posterior 4‐ to 10‐mm margins. In IMRT, PTV‐local was defined as CTV‐local with 5‐ to 8‐mm margins except for the posterior rectal side with 4‐ to 6‐mm margins. In LN boost, CTV‐LN was defined as metastatic LN with 0‐ to 5‐mm margins. PTV‐LN was defined as CTV‐LN with 0‐ to 5‐mm margins considering how close bowel was located to CTV‐LN.

The dose escalation to metastatic LN was performed with various doses and fractionations using IMRT. The RT dose to metastatic LN was escalated using simultaneous integrated boost (SIB) technique. The RT dose to metastatic LN was not fixed but escalated as much as dose constraints for organs at risk, especially bowel surrounding LN allows. We calculated the metastatic LN dose using the equivalent dose in 2 Gy fractions (EQD2), according to the Linear‐Quadratic model.^12^ Because the α/β ratio of prostate cancer usually assumed to be low, we set α/β ratio as 1.5 in the present study.^13^

### 2.3 Statistical analysis

We calculated the overall survival (OS), cause‐specific survival (CSS), relapse‐free survival (RFS), distant metastasis‐free survival (DMFS), and biochemical relapse‐free survival (BRFS). For CSS, death of patients with prostate‐specific antigen (PSA) relapse or distant metastases was defined as death due to prostate cancer. Patients who died from causes unrelated to prostate cancer were not counted in CSS. RFS included local‐regional or distant recurrence as diagnosed by clinical exam, imaging, and/or biopsy. In DMFS calculation, death without distant metastasis was censored. Bone scintigraphy and CT were performed to detect distant metastases. Pelvic LN metastases were not included in distant metastases but in local‐regional metastases. BRFS was calculated from the start of radiotherapy to PSA relapse as an event. Death without PSA relapse was considered as censored in BRFS. PSA relapse was defined using Phoenix definition (nadir plus 2 ng/mL).^14^

Acute and chronic toxicities were assessed according to the Common Terminology Criteria for Adverse Events version 4.0. Chronic toxicity was defined as toxicity seen more than 3 months after completion of radiotherapy.

Our routine follow‐up included PSA measurements every 3‐6 months after radiotherapy. If PSA relapse occurred, restaging scans, such as clinical exam, imaging, and/or biopsy, were done for detecting local‐regional or distant recurrence in some patients, and hormonal therapy was started in other patients without restaging scans. Patients lost to follow‐up were censored at the time of the last follow‐up observation. Survival curves were drawn using the Kaplan‐Meier estimator. Univariate analyses using log‐rank test were performed to find prognostic factors between patient subgroups [age at RT, pretreatment PSA, T stage, Gleason score (GS), local and LN RT dose, and ADT duration period] in OS, CSS, RFS, DMFS, and BRFS. The Chi‐square test was used to compare the incidence rate of adverse events by each RT dose. The tests were two‐sided, and statistical significance was set at *P* < .05. Statistical analysis was performed using JMP 14 (SAS Institute Inc).

## 3 RESULTS

We identified 51 consecutive cN1 patients who underwent definitive pelvic RT between June 2004 and February 2016. Their clinicopathologic characteristics are summarized in Table 1. Advanced T stage (3 or more) was diagnosed in 45 (88%) patients. Median initial PSA was 48 (range 4.6‐531) ng/mL. GS 8 or more and primary Gleason pattern (GP) 5 were seen in 32 (63%) and eight (16%) patients, respectively. All patients received ADT.

**TABLE 1:** Clinicopathologic characteristics of patients

Characteristic	n = 51
Age (y)	67 (range 51‐83)
Median initial PSA (range), ng/mL	48 (range 4.6‐531)
Clinical stage, n (%)	
Missing	3 (5.9)
T1	3 (5.9)
T2	0 (0)
T3	37 (73)
T4	8 (16)
Gleason score, n (%)	
3+3	0 (0)
3+4	3 (5.9)
4+3	16 (31)
4+4	1 (2.0)
4+5	23 (45)
5+4	6 (12)
5+5	2 (3.9)
ADT, n (%)	
Yes	51 (100)
No	0 (0)

Abbreviations: ADT, androgen deprivation therapy; PSA, prostate‐specific antigen.

The treatment characteristics are summarized in Table 2. The median follow‐up period for all patients was 88 (range 20‐157) months. The method of RT was IMRT only in 23 (45%) patients, 3DCRT only in 19 (37%) patients, combined IMRT and 3DCRT in eight (16%) patients, and combined high‐dose rate brachytherapy (HDR) and IMRT in one (2%) patient. We performed LN dose escalation for some patients who received IMRT. Twenty‐three patients (45%) received 60 Gy or more of LN RT dose.

**TABLE 2:** Treatment Characteristics

Characteristics	
Median follow‐up period (range), month	88 (20‐157)
Radiation method, n (%)	
3DCRT	19 (37)
3DCRT+IMRT	8 (16)
HDR+IMRT	1 (2)
IMRT	23 (45)
Median radiation dose (range)	
Whole pelvic LN area, Gy	46 (46)
Positive LNs (Dmean of GTVn), Gy	46.7 (46‐72)
Positive LNs (Dmean of GTVn), BED(α/β 1.5)	110 (107‐195)
Prostate + Seminal vesicle, Gy	72 (66‐78)
Median duration period of ADT (range), month	19 (3‐45)

Abbreviations: 3DCRT, three‐dimensional conformal radiation therapy; ADT, androgen deprivation therapy; HDR, high‐dose rate brachytherapy; IMRT, intensity‐modulated radiation therapy; LN, lymph node.

Biochemical recurrence was seen in 27 (53%) patients, 15 (29%) of whom underwent clinical recurrence. Loco‐regional recurrence was seen in five (10%) patients, three in local region (prostate or seminal vesicle), one in pelvic LN, and one in both local region and pelvic LN. Distant recurrence was seen in 15 (29%) patients, 12 in bone, one in muscle, one in brain, and one in liver, respectively.

Among the patients who underwent biochemical recurrence, four patients underwent restaging scans when they developed biochemical recurrence, five patients when some symptoms such as pain occurred, and five patients when hormonal therapy became refractory. Hormonal therapy or observation without restaging scans was done in the other patients, nine of whom underwent diagnostic imaging as examinations of the other diseases.

**FIGURE 1:**
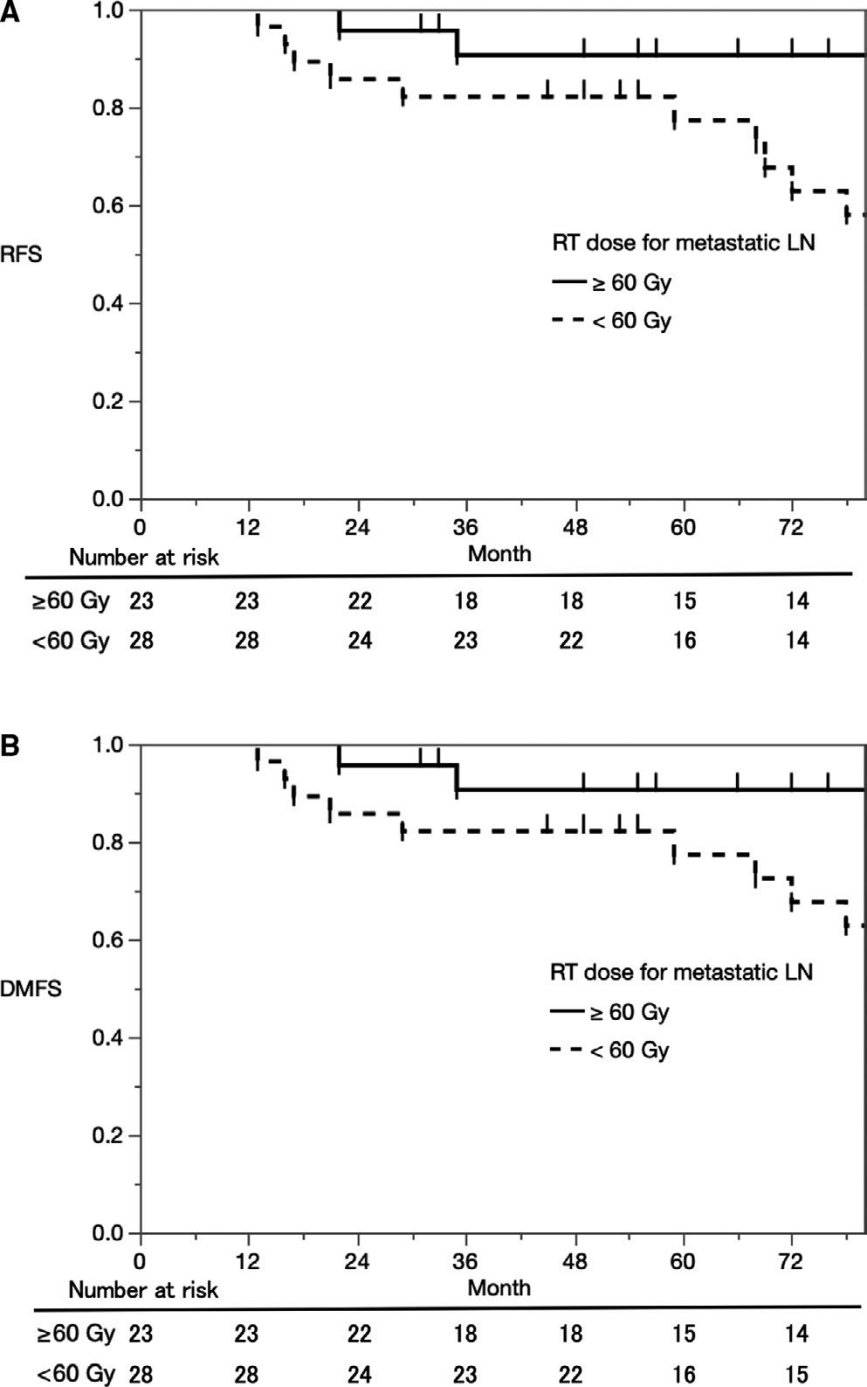
A, Kaplan‐Meier estimates for comparison of relapse‐free survival (RFS) between higher lymph node (LN) RT dose (60 Gy or more) group and lower LN RT dose (less than 60 Gy) group. B, Kaplan‐Meier estimates for comparison of distant metastasis‐free survival (DMFS) between higher lymph node (LN) RT dose (60 Gy or more) group and lower LN RT dose (less than 60 Gy) group

Results of univariate analyses are summarized in Table 3. Primary GP was the only statistically significant prognostic factor for OS and CSS. Primary GP and LN RT dose were the statistically significant prognostic factors for RFS and DMFS. Especially, higher RT dose (60 Gy or more) for metastatic LN significantly improved RFS (4‐year RFS 90.6% vs 82.1%, 7‐year RFS 90.6% vs 58.0%, *P* = .015) (Figure 1A) and DMFS (4‐year DMFS 90.6% vs 82.1%, 7‐year DMFS 90.6% vs 62.8%, *P* = .023) (Figure 1B). T stage, LN RT dose, local RT dose, and ADT duration period were statistically significant for BRFS.

**TABLE 3:** Results of univariate analyses of OS, CSS, RFS, DMFS, and BRFS

Factor	n	4‐y OS rate (%)	*P*	4‐y CSS rate (%)	*P*	4‐y RFS rate (%)	*P*	4‐y DMFS rate (%)	*P*	4‐y BRFS rate (%)	*P*
		7‐y OS rate (%)		7‐y CSS rate (%)		7‐y RFS rate (%)		7‐y DMFS rate (%)		7‐y BRFS rate (%)	
T stage											
1, 2, 3a	17	100	.920	100	.363	86.9	.118	86.9	.130	71.8	.035*
		82.1		100		86.9		86.9		71.8	
3b, 4	23	95.7		95.7		82.6		82.6		47.1	
		84.8		84.8		64.9		70.8		35.3	
Unknown	11	NA		NA		NA		NA		NA	
iPSA											
<50	25	95.7	.289	90.6	.729	73	.720	73	.720	51.8	.896
		78.2		74.4		73		73		51.8	
≥50	23	96		96		96		96		67.8	
		85.3		77.3		78.5		78.5		37.8	
Unknown	3	NA		NA		NA		NA		NA	
Gleason score											
<8	19	100	.678	100	.692	94.1	.140	94.1	.143	64.4	.188
		90.9		100		86.3		94.1		57.2	
≥8	32	93.8		93.8		81.3		81.3		55.3	
		78.7		85.9		63.9		63.9		31.8	
Primary Gleason pattern											
5	8	97.7	.002*	97.7	.002*	90.5	.021*	90.5	.012*	62.7	.226
		91.2		97.7		77.4		80.7		40.8	
3,4	43	87.5		87.5		62.5		62.5		37.5	
		46.9		56.3		41.7		41.7		37.5	
LN RT dose (EQD2)											
≥60 Gy	23	100	.236	100	.739	90.6	.015*	90.6	.023*	70.7	.035*
		95		95		90.6		90.6		59.8	
<60 Gy	28	92.9		92.9		82.1		82.1		50	
		74.9		87.7		58		62.8		25.6	
Local RT dose											
>72 Gy	18	100	.793	100	.721	87.7	.275	87.7	.293	80.8	.006*
		92.9		92.9		87.7		87.7		80.8	
≤72 Gy	33	93.9		93.9		84.9		84.9		48.5	
		80.5		90.3		67.9		71.3		26.3	
Duration of ADT											
≥12 mo	34	94.1	.373	94.1	.651	85.3	.908	85.3	.766	69.9	.0236*
		86.7		86.7		72.3		72.3		49.3	
<12 mo	17	100		100		87.4		87.4		33.6	
		77.9		100		71.5		79.5		20.2	

Abbreviations: ADT, androgen deprivation therapy; BRFS, biochemical relapse‐free survival; CSS, cause‐specific survival; DMFS, distant metastasis‐free survival; LN, lymph node; OS, overall survival; PSA, prostate‐specific antigen; RFS, relapse‐free survival; RT, radiotherapy.

* *P* < .05.

Acute and chronic toxicities for local RT dose, LN RT dose, and RT method are summarized in Table 4. Acute adverse toxicities of grade 3 or greater were not observed in any group. Among chronic genitourinary toxicities, one patient developed grade 3 hematuria and one developed grade 3 urinary retention. The patient with chronic grade 3 hematuria belonged to lower LN RT dose (less than 60 Gy) group and lower local RT dose (72 Gy or less) group and underwent hyperbaric oxygen therapy. The patient with chronic grade 3 urinary retention belonged to higher LN RT dose (60 Gy or more) group and lower local RT dose (72 Gy or less) group and developed acute renal failure that improved with urethral catheterization. Chronic gastrointestinal toxicities of grade 3 or greater were not observed in any group.

**TABLE 4:** Incidence of toxicities (n [%]) according to the common terminology criteria for adverse events ver. 4

	LN RT dose (EQD2)					Local RT dose (EQD2)					Method of RT					
	≥G2			G3		≥G2			G3		≥G2			G3		
	≥60 Gy	<60 Gy	*P*	≥60 Gy	<60 Gy	>72 Gy	≤72 Gy	*P*	>72 Gy	≤72 Gy	3DCRT	3DCRT + IMRT	IMRT	3DCRT	3DCRT + IMRT	IMRT
Acute																
Urinary frequency	20 (87)	19 (68)	.110	0 (0)	0 (0)	14 (78)	25 (76)	.871	0 (0)	0 (0)	13 (68)	6 (75)	19 (83)	0 (0)	0 (0)	0 (0)
Urinary retention	15 (65)	6 (21)	.002*	0 (0)	0 (0)	8 (44)	13 (39)	.726	0 (0)	0 (0)	3 (16)	5 (63)	12 (52)	0 (0)	0 (0)	0 (0)
Nausea	1 (4.4)	0 (0)	.265	0 (0)	0 (0)	1 (5.6)	0 (0)	.171	0 (0)	0 (0)	0 (0)	0 (0)	1 (4.3)	0 (0)	0 (0)	0 (0)
Diarrhea	12 (52)	14 (50)	.877	0 (0)	0 (0)	9 (50)	17 (52)	.918	0 (0)	0 (0)	12 (63)	4 (50)	10 (43)	0 (0)	0 (0)	0 (0)
Chronic																
Hematuria	2 (8.7)	4 (14)	.538	0 (0)	1 (3.6)	2 (11)	4 (12)	.915	0 (0)	1 (3.0)	3 (16)	1 (13)	2 (8.7)	1 (5.3)	0 (0)	0 (0)
Urinary retention	2 (8.7)	1 (3.6)	.439	1 (4.3)	0 (0)	1 (5.6)	2 (6.1)	.942	0 (0)	1 (3.0)	0 (0)	1 (13)	1 (4.3)	0 (0)	1 (13)	0 (0)
Urinary incontinence	4 (17)	4 (14)	.762	0 (0)	0 (0)	1 (5.6)	7 (21)	.142	0 (0)	0 (0)	3 (16)	3 (38)	2 (8.7)	0 (0)	0 (0)	0 (0)
Rectal hemorrhage	3 (13)	5 (18)	.638	0 (0)	0 (0)	4 (22)	4 (12)	.343	0 (0)	0 (0)	4 (21)	1 (13)	3 (13)	0 (0)	0 (0)	0 (0)

Abbreviations: 3DCRT, three‐dimensional conformal radiation therapy; IMRT, intensity‐modulated radiation therapy; LN, lymph node; RT, radiotherapy.

* *P* < .05.

Any significantly different toxicity was not seen for each LN or local RT dose, except for the incident rate of grade 2 or more acute urinary retention, which was significantly higher in the higher LN RT dose (60 Gy or more) group by the Chi‐square test (Table 4). There is no significant difference in toxicity between IMRT group and 3DCRT group (Table 4).

## 4 DISCUSSION

NCCN classification recognizes cN1 as a regional disease.^1^ Several studies have shown that addition of local treatment (surgery and RT) to ADT improves treatment outcome.^2^, ^3^, ^4^, ^5^, ^6^, ^7^, ^8^, ^9^ Thus, in the NCCN guideline 2018, external beam RT combined with ADT ± abiraterone and prednisone is regarded as one of the standard therapies along with ADT ± abiraterone and prednisone.^1^ The guideline further recommends nodal radiation and dose escalation to clinically positive nodes considering the tolerance dose of surrounding tissue. In 2019 Recommendations of the Australian and New Zealand Radiation Oncology Genito‐Urinary group, it is recommended that gross nodal disease should be treated by a higher dose of more than 60 Gy while maintaining safe, normal tissue dose constraints for cN1 patients.^15^ Although RT for cN1 is being recognized as a standard therapy, the method of RT has not been examined enough. For localized lesions (prostate and/or seminal vesicle), the benefit of dose escalation has been demonstrated.^16^, ^17^, ^18^, ^19^, ^20^, ^21^ However, the benefit of dose escalation to clinically positive pelvic LN has not yet been demonstrated. To the best of our knowledge, this study is the first to compare the different RT dose to clinically positive nodes and report the benefits of dose escalation to metastatic pelvic nodes in cN1 patients.

There are several retrospective or database studies which demonstrate the benefits of RT for pathologically (p) or clinically (c) N1 patients. Zagars et al retrospectively showed that local RT combined with ADT, after staging lymphadenectomy, significantly improved disease control and patient survival compared with ADT alone in pN1 patients.^2^ Kuefer et al retrospectively showed that both RP and RT to prostate benefited the pN1 and cN1 patients.^3^ Abdollah et al retrospectively showed that adjuvant RT after RP and extended pelvic LN dissection, improved CSS in pN1 patients.^4^ The RT group showed better OS and CSS compared with no local treatment group among N1 patients based on analysis of the Surveillance, Epidemiology, and End Results database.^6^ The cN1 patients in The National Cancer Database of USA had a significant survival benefit when treated with ADT + RT compared with ADT alone.^7^ Although these studies clearly demonstrate the benefits of RT, the radiation fields or prescribed doses of RT are not well considered.

Three nonrandomized studies have utilized higher elective nodal doses.^22^, ^23^, ^24^ Adkinson et al prescribed 70 Gy to prostate and 56 Gy to nodal region in 28 fractions and they demonstrated the feasibility of the schedule in median follow‐up period of 25.4 months.^22^ Engels et al utilized helical tomotherapy for 28 patients who were treated to a dose of 54 Gy in daily fractions of 1.8 Gy to the pelvic lymph node area, while the prostate and the seminal vesicles received a simultaneous integrated boost (SIB) to a dose of 70.5 Gy. A SIB to a dose of 60 Gy was delivered to the involved lymph node region(s) in eight patients with pelvic lymph node metastases. They concluded that pelvic nodal dose escalation in node‐positive patients is feasible without increasing toxicity.^23^ Fonteyne et al reported late toxicity at 3 years. They treated the prostate to a median dose of 69.3 Gy, elective nodes to a minimal dose of 45 Gy, and gross nodal disease to 65 Gy in 25 fractions using a simultaneous integrated boost.^24^ In the present study, though we irradiated the whole pelvic LN area for all patients, the prescribed doses for positive LN or local region were different for each patient. We compared clinical outcomes by differences in RT dose to local and positive lymph nodes.

Higher local RT dose (more than 72 Gy) significantly improved BRFS in the present study. Our results are similar to previous studies. Several randomized controlled trials have shown improved failure‐free survival with higher local RT dose for localized prostate cancer.^16^, ^17^, ^19^, ^20^ In the database study, higher local RT dose improved overall survival (OS) in intermediate‐ and high‐risk localized prostate cancer patients.^21^

NCCN guideline 2018 recommends nodal radiation and dose escalation to clinically positive nodes considering the tolerance dose of surrounding tissue.^1^ In Recommendations of the Australian and New Zealand Radiation Oncology Genito‐Urinary group 2019, it is recommended that gross nodal disease should be treated by a higher dose of more than 60 Gy while maintaining safe normal tissue dose constraints for cN1 patients.^15^ In this study, because dose escalation to 60 Gy seems to be feasible considering surrounding normal organ, especially bowel, we divided patients into higher LN dose and lower LN dose group by 60 Gy. Higher LN RT dose (60 Gy or more) significantly improved BRFS, RFS, and DMFS compared with lower LN RT dose (less than 60 Gy) in the present study. However, higher LN RT doses did not improve OS and CSS. Pollack et al reported that biochemical failure, as a time‐dependent covariate, was the strongest determinant of distant metastasis and was also very significantly related to cause‐specific death.^25^ Even if the patients develop distant metastasis, it will be a long time for the death caused by prostate cancer because of the effective treatment of ADT and/or other agents.^1^ Therefore, in the present study, we consider that it takes longer time until the improvement of RFS and DMFS results in the improvement CSS and OS.

We found that primary GP is only the significant prognostic factor for both OS and CSS. This is consistent with the NCCN guidelines 2018, in which primary GP 5 is considered a poor prognostic factor and classified as very high‐risk group.^1^

Even though higher dose RT improves survival, there is concern that it may increase the incidence of adverse events. Dose escalation to local region has increased adverse events, especially late gastrointestinal toxicity.^19^ In the present study, severe toxicities, grade 3 or more, were seldom observed in both higher and lower local or LN dose group. Thus, dose escalation, especially to positive LN, seemed to be safe in the present study. However, because the present study was a single institutional retrospective study, there is a concern about the robustness of the adverse events. Further multicenter, prospective studies with larger sample size are warranted.

There are several limitations to our study. First, this study is a retrospective study from a single institution, which could introduce some unknown biases. Second, there are a limited number of patients in this study, because of which a multivariate analysis was not possible. Third, the RT method changed from 3DCRT to IMRT in the study period. IMRT changed not only RT dose but also RT field and require highly sophisticated technique of image‐guided radiotherapy, which could unexpectedly affect treatment outcomes. Therefore, we recommend multicenter, prospective studies with larger sample size to confirm the findings.

## 5 CONCLUSION

RT dose escalation to positive pelvic nodes in cN1 patients could improve RFS and DMFS at 4 and 7 years, without increasing severe adverse events. Large‐scale, prospective studies are warranted to definitively confirm our findings.

## CONFLICT OF INTEREST

All authors have no conflict of interest to declare.

## AUTHOR CONTRIBUTIONS

Keisuke Tsuchida collected and analyzed the data and drafted the manuscript. Tairo Kashihara, Naoya Murakami, Kae Okuma, Kana Takahashi, Hiroshi Igaki, Yuko Nakayama, Aiko Maejima, Yasuo Shinoda, Yoshiyuki Matsui, Motokiyo Komiyama, Hiroyuki Fujimoto, Yoshinori Ito, Minako Sumi, and Jun Itami collected the data. Koji Inaba aided in writing manuscript and contributed to the final draft of the manuscript. Takashi Nakano and Jun Itami contributed to the final draft of the manuscript. All authors read and approved the final manuscript.

## ETHICS APPROVAL

This retrospective study was approved by the Institutional Ethical Review Board of the National Cancer Center Hospital (2017‐091), and was performed in accordance with the ethical standards laid down in the 1964 Declaration of Helsinki and its later amendments. Written informed consent to treatment was taken from all the participants included in this study before treatment.

## Data Availability

Research data are not shared.
